# Association of gestational diabetes mellitus with offspring weight status across infancy: a prospective birth cohort study in China

**DOI:** 10.1186/s12884-020-03494-7

**Published:** 2021-01-06

**Authors:** Jiajin Hu, Yilin Liu, Xiaotong Wei, Lin Li, Ming Gao, Yang Liu, Yanan Ma, Deliang Wen

**Affiliations:** 1 Institute of Health Sciences, China Medical University, Shenyang, 110122 Liaoning China; 2 Research Center of China Medical University Birth Cohort, China Medical University, Shenyang, 110122 Liaoning China; 3 Division of Chronic Disease Research Across the Lifecourse, Department of Population Medicine, Harvard Medical School, Boston, MA 02215 USA; 4 Department of Epidemiology and Health Statistics, School of Public Health, China Medical University, Shenyang, 110122 Liaoning China

**Keywords:** Pregnancy, Gestational diabetes mellitus, Infancy, Obesity, Birth cohort

## Abstract

**Background:**

The association between gestational diabetes mellitus (GDM) and childhood body weight remains controversial, and additional study is needed, especially in Asian populations.

**Methods:**

This prospective study investigated the association between maternal glucose concentration, and GDM status and infant body weight from birth to 12 months of age. Linear mixed effects (LME) models and multiple linear regression were used to assess the longitudinal association of GDM with infant growth measured by weight-for-length z-scores (WFLZ), weight-for-age z-scores (WFAZ), and length-for-age z-scores (LFAZ) at birth, 1, 3, 6, 8, and 12 months of age.

**Results:**

Offspring born to mothers with GDM had higher WFLZ [β: 0.26 SD units (95% CI: 0.13–0.40)] across infancy than those of mothers without GDM. When stratified analysis by maternal pre-pregnancy body mass index (BMI) status, the association was pronounced in normal-weight [β:0.28 SD units (95% CI: 0.11–0.45)] and overweight/obese women [β: 0.34 SD units (95% CI: 0.09–0.58)] but not in underweight women (*P* for interaction < 0.05). Multiple linear regression found that the effect estimate of GDM on infant WFLZ was highest at birth [β: 0.36 SD units (95% CI: 0.11–0.61)], remained significant at 1 [β: 0.22 SD units (95% CI: 0.03–0.41)] and 3 [β:0.19 SD units (95% CI: 0.01–0.37)] months of age and decreased across infancy. Maternal GDM status was not associated with infant WFAZ or LFAZ.

**Conclusions:**

Maternal GDM status was associated with infant WFLZ, but not WFAZ or LFAZ. The association between GDM status and offspring WFLZ was more pronounced in early infancy or in normal-weight and overweight/obese women. Increased public health efforts to prevent GDM in normal-weight and overweight/obese pre-pregnancy mothers are recommended to control offspring overweight or obesity.

**Supplementary Information:**

The online version contains supplementary material available at 10.1186/s12884-020-03494-7.

## Background

The continuing increase in the prevalence of childhood obesity has become a global public health challenge [1]. Gestation has been identified as a critical time for adiposity programing of offspring [2]. Exposure to metabolic changes associated with gestational diabetes mellitus (GDM) may alter the in utero environment to increase the risk of obesity in later life. Previous studies have found an association between GDM and increased birth weight, but the evidence for an association between GDM and childhood overweight is inconsistent [3–7]. Some studies found that maternal hyperglycemia was associated with an increased risk of childhood overweight/obesity, but others did not [8–26]. The in utero effect of GDM on offspring may be time sensitive and may also vary with the age of the child [5, 11, 20, 27, 28]. More studies are needed to investigate the long-term effect of GDM on the growth trajectory and risk of obesity in children.

The effects of maternal hyperglycemia on the programming of childhood obesity may vary among races [29]. Asian populations are thought to be at higher risk of GDM than western populations [30, 31]. In China, the prevalence of GDM is estimated to range from 9.3 to 18.9% depending on the region [32–34]. Only two studies have investigated the effect of GDM on childhood obesity, and the results are conflicting [35, 36]. A large study by Liu et al. reported that GDM was associated with an increased weight-for-length z-score (WFLZ) at birth but not later in life [35]. Zhao et al. found that the birth weights of children born to mothers with GDM and without GDM were not significantly different, but that the body mass index (BMI) and z-scores of those born to mothers with GDM were higher at 12 months after birth [36]. The influence of GDM on offspring weight status may be influenced by the mother’s pre-pregnancy BMI status. Previous studies of the association between GDM and childhood obesity have included maternal pre-pregnancy BMI as a covariate [5, 6]. To our knowledge, no previous study has investigated the correlation between GDM and childrens’ weight status according to maternal pre-pregnancy BMI status [37, 38], which would contribute to improved characterization of the target population for interventions to prevent childhood obesity.

To better define maternal targets for interventions to prevent childhood obesity, this study investigated the association between maternal hyperglycemia and GDM status and infant weight status from birth to 1 year of age using data obtained in the Born in Shenyang Birth Cohort Study (BISCS) [39]. We hypothesize that:

1. Maternal hyperglycemia and GDM will be correlated with increased infant body weight.
2. The association between GDM and infant weight would vary with maternal pre-pregnancy BMI status.

## Methods

### Study population and design

The study included mothers and infants who had participated in the Born in Shenyang Cohort Study, a prospective, observational cohort study of gestational factors, pregnancy outcomes, and offspring health. The study design and the description of the interview has been published elsewhere [39]. Briefly, the BISCS was a prospective study that enrolled healthy women with single pregnancies at 21–24 weeks gestation at 54 hospitals or community healthcare centers in the urban area of Shenyang, northeast China between April and September 2017. Of a cohort of 2068 women who were invited to participate, 1338 (mean gestation of 22 ± 1.2 weeks) agreed, and 1290 of them had live singleton births. Research fellows conducted home visits with mothers and infants within 7 days of birth and at visits to child development clinics at 1, 3, 6, 8, and 12 months of age. Follow-up data were available from 72.4% of the 1290 mothers and their infants at 1 month, 71.2% at 3 months, 66.7% at 6 months, 77.8% ant 8 months, and 77.4% at 12 months.

Trained research fellows conducted face-to-face interviews at enrollment and follow-up visits. Demographic, societal, and clinical characteristics of the mothers were collected by a structured questionnaire developed for this study (Additional File 6). Anthropometric measurements of the infants were performed at each follow-up visit. Among 1290 mother–infant pairs, 210 with missing oral glucose tolerance test (OGTT) results, 133 with missing anthropometric data, and two with pregestational diabetes mellitus were excluded. The remaining 945 mother–infant pairs were included in the final analysis, and the results were compared with the observations in the 345 excluded participants. The characteristics of the two groups were similar.

### Gestational Diabetes mellitus

GDM was diagnosed when at least one of the following criteria was met during a 75 g OGTT at 24–28 weeks of pregnancy: a fasting plasma glucose of ≥5.1 mmol/L, a 1-h plasma glucose of ≥10.0 mmol/L, or a 2-h plasma glucose of ≥8.5 mmol/L [40]. Pregnant women were assigned to either a GDM or a non-GDM group based on the diagnosis.

### Anthropometric characteristics of infants

Birth weight and length were derived from medical records. Infant weight and length were measured by trained research fellows at 1, 3, 6, 8, and 12 months of age with calibrated infant stadiometers (Seca 416; Seca Corporation, Hamburg, Germany) and weighing scales (Seca 376+). The mean values of two successive measurements were reported. Sex- and length-specific z-scores for weight (WFLZ) and sex- and age-specific z-scores for BMI (BMIZ), weight (WFAZ), and length (LFAZ) were calculated using the World Health Organization child growth references [41]. Infant overweight/obesity was defined as a WFLZ of > 2 according to the WHO standard [41].

### Covariates

At the enrollment visit, participants self-reported their age, ethnicity, educational attainment, income level, parity, gestational age, and pre-pregnancy weight using standard questionnaires. Height was measured with a calibrated stadiometer. Age in years and gestational age in weeks were analyzed as continuous variables. The mother’s ethnicity was recorded as either Han or other. Women were categorized by educational level to four groups (middle school or below, high school, college, graduate school or above) and by parity to either one or more than one pregnancies. Maternal pre-pregnancy and paternal BMIs were categorized to three groups, [underweight (BMI < 18.5 kg/m^2^) vs. normal weight (18.5 kg/m^2^ ≤ BMI < 24 kg/m^2^) vs. overweight/obese (BMI ≥24 kg/m^2^)] using Chinese reference values [42]. Overweight and obese women were included in a single category because of the limited sample size.

### Statistical analysis

The longitudinal associations between GDM status, i.e., with or without GDM, and blood glucose concentration (mmol/L) and infant WFLZ, WFAZ, and LFAZ from birth to 12 months were determined using linear mixed effects (LME) models. LME takes into account within-subject correlation of repeated measurements and also compensates for incomplete outcome measurements. The models included an unstructured covariance matrix for random-effect variables (intercept and slope) and a maximum likelihood-estimation method. Crude and adjusted analyses were performed with three models. Model 1 was adjusted for linear, quadratic and, cubic terms for infant’s age to estimate the association between GDM and blood glucose concentration with z-scores growth estimates across infancy. In model 2, we further adjusted for maternal pre-pregnancy BMI, as it was associated with infant overweight/obesity status. In model 3, we further adjusted covariates associated with GDM status, including maternal age, parity and gestational age. Stratified analyses were performed to investigate the association between GDM status and infant size in women of different pre-pregnancy BMI status (underweight vs. normal weight vs. overweight/obese). Interactions of GDM status and blood glucose level with pre-pregnancy BMI category and their association with infant growth measurements were tested by including the corresponding interactions into the models. The association between GDM status and blood glucose level and infant sex- and age-specific z-scores for WFL, weight and length at birth and at 1, 3, 6, 8, and 12 months of age was estimated by multivariable linear regression. The full model was adjusted for pre-pregnancy BMI, maternal age, parity and gestational age. Multiple imputation was performed to compensate for missing values in the linear regression models. In the sensitivity analysis, BMIZ was compared with WFLZ as outcomes of the LME analysis (Table S2). To assess the robustness of the study findings, all analyses were repeated in participants without missing covariate or outcomes data (*n* = 524, Table S3) or repeated in models adjusted only for maternal pre-pregnancy BMI (Fig. S1 and S2). The statistical analysis was performed with Stata/SE version 13 (StataCorp, College Station, TX, USA). Two-sided *p*-values < 0.05 were considered statistically significant.

## Results

### Participant characteristics

Differences in demographic (maternal ethnicity, education level, or annual household income) or clinical characteristics (paternal BMI, infant sex) in the GDM and non-GDM groups were not significant. Women with GDM were more likely to be older (31.3 vs. 29.8 years of age), have a shorter gestation at delivery (38.6 vs. 38.9 weeks), a lower proportion of nulliparity (69.3% vs. 79.1%), and were more likely to be overweight before pregnancy (35.6% vs. 22.7%) than women without GDM (Table 1). Infants who were overweight/obese at 1-year of age were more likely to be male or have mothers with a pre-pregnancy BMI of ≥24.0 kg/m^2^ (Table S1).

**Table 1:** Characteristics of mothers with and without gestational diabetes mellitus

Characteristics	Gestational Diabetes Mellitus, Mean ± SD or n (%)		
	No	Yes	***P***-value
	***n*** = 740 (78%)	***n*** = 205 (22%)	
Age at enrollment (Years)	29.8 ± 3.8	31.3 ± 4.2	< 0.001
Ethnicity			0.72
Han	613 (82.8)	127 (17.1)	
Others	172 (83.9)	33 (16.1)	
Educational attainment			0.64
Middle school or below	60 (8.1)	17 (8.3)	
High school	107 (14.5)	31 (15.1)	
College	507 (68.5)	133 (64.9)	
Graduate or above	66 (8.9)	24 (11.7)	
Household income per year, CNY			0.83
< 30,000	197 (26.6)	51 (24.9)	
30,000- < 50,000	191 (25.8)	57 (27.8)	
50,000- < 70,000	166 (22.4)	42 (20.5)	
≥ 70,000	186 (25.1)	55 (26.8)	
Parity			0.003
1	585 (79.1)	142 (69.3)	
> 1	155 (21.0)	63 (30.7)	
Gestational age (Weeks) ^a^	38.9 ± 1.3	38.6 ± 1.2	< 0.001
Pre-pregnancy BMI category, kg/m^2^			< 0.001
< 18.5	121 (16.4)	12 (5.9)	
18.5- < 24.0	451 (61.0)	120 (58.5)	
≥ 24.0	168 (22.7)	73 (35.6)	
Paternal BMI category, kg/m^2 b^			0.27
< 18.5	22 (3.1)	2 (1.0)	
18.5- < 24.0	274 (38.3)	74 (37.6)	
≥ 24.0	419 (58.6)	121 (61.4)	
Infant sex			0.41
Male	356 (48.1)	92 (44.9)	
Female	384 (51.9)	113 (55.1)	

*CNY* Chinese Yuan, *BMI* body mass index

^a^: *n* = 942; ^b^: *n* = 912 because of missing data

### Longitudinal analysis (LME models)

Table 2 shows the LME model results of the association between GDM status and blood glucose levels and infant growth z-scores. After adjusting for sociodemographic characteristics and parental BMI, the infants of mothers with GDM had higher WFLZ scores [β:0.26 SD units (95% CI: 0.13–0.40)] than those without GDM. Increased fasting blood glucose concentration was associated with increased infant WFLZ [β:0.12 SD units (95% CI: 0.01–0.23), but the 1 h and 2 h OGTT glucose concentrations were not. GDM status and blood glucose concentration were not associated with either WFAZ or WFLZ. In the sensitivity analysis, use of BMIZ or WFLZ as the outcome, or limiting the analysis to mothers and infants with complete data (*n* = 524) did not change the results (Tables S2 and S3).

**Table 2:** Association of GDM status and blood glucose level with infant growth measures using linear mixed effects model

Infant Growth Measures	GDM (ref. = non-GDM), β (95% CI)	Blood Glucose Level, β (95% CI)		
		Fasting	1 h after OGTT	2 h after OGTT
WFLZ				
Model 1 ^a^	0.29 (0.15, 0.42)	0.14 (0.04, 0.25)	0.03 (0.00, 0.06)	0.02 (−0.02, 0.07)
Model 2 ^a^	0.25 (0.12, 0.38)	0.11 (0.00, 0.21)	0.00 (−0.03, 0.03)	0.02 (− 0.03, 0.06)
Model 3 ^b^	0.26 (0.13, 0.40)	0.12 (0.01, 0.23)	0.03 (−0.01, 0.06)	0.02 (− 0.02, 0.06)
WFAZ				
Model 1 ^a^	0.13 (0.00, 0.26)	0.13 (0.02, 0.23)	0.11 (−0.02, 0.04)	0.02 (−0.02, 0.06)
Model 2 ^a^	0.09 (−0.04, 0.23)	0.09 (−0.01, 0.20)	0.00 (− 0.03, 0.04)	0.01 (− 0.03, 0.05)
Model 3 ^b^	0.13 (0.01, 0.26)	0.13 (0.03, 0.23)	0.02 (−0.02, 0.05)	0.03 (−0.01, 0.06)
LFAZ				
Model 1 ^a^	−0.09 (− 0.24, 0.06)	0.04 (− 0.08, 0.16)	−0.01 (− 0.05, 0.03)	−0.02 (− 0.07, 0.02)
Model 2 ^a^	− 0.10 (− 0.26, 0.05)	0.03 (− 0.09, 0.15)	−0.01 (− 0.05, 0.03)	−0.03 (− 0.07, 0.02)
Model 3 ^b^	− 0.06 (− 0.20, 0.08)	0.08 (− 0.04, 0.18)	0.00 (− 0.03, 0.03)	−0.01 (− 0.06, 0.03)

Model 1: adjusted for the age of infants at each measurement

Model 2: Model 1 + pre-pregnancy BMI

Model 3: Model 2 + maternal age, parity, gestational age

*Abbreviations: GDM* gestational diabetes mellitus, *CI* confidence interval, *OGTT* oral glucose tolerance test, *WFLZ* weight-for-length z-score, *WFAZ* weight-for-age z-score, *LFAZ* length-for-age z-score, *BMI* body mass index

^a^: *n* = 945; ^b^: *n* = 942 because of missing data

### Association between GDM and infant WFLZ after stratification by maternal pre-pregnancy weight

**Fig. 1:**
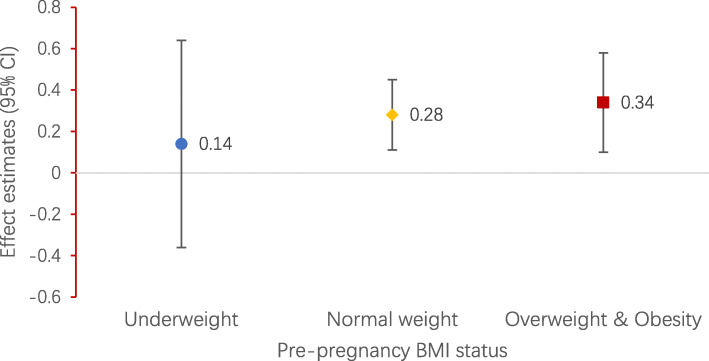
Association between GDM (ref. = non-GDM) with infant sex-specific WFLZ (β, 95% CI) from birth to 12 months by pre-pregnancy weight status. Models are adjusted for exact age of children at each measurement, pre-pregnancy BMI, maternal age, parity, gestational age. Abbreviations: GDM, gestational diabetes mellitus; CI, confidence interval; WFLZ, weight-for-length z-score; BMI, body mass index

Stratification by pre-pregnancy weight resulted in significant differences in the association between GDM and infant WFLZ (*P* for interaction < 0.05). GDM was associated increased infant WFLZ in normal-weight [β:0.28 SD units (95% CI: 0.11–0.45)] and in overweight/obese women [β: 0.34 SD units (95% CI: 0.09–0.58)]. GDM was not associated infant WFLZ in underweight women (Fig. 1). In the sensitivity analysis, adjusting for pre-pregnancy BMI did not significantly change the results (Fig. S1).

### GDM status and infant growth from birth through 12 months of age

**Fig. 2:**
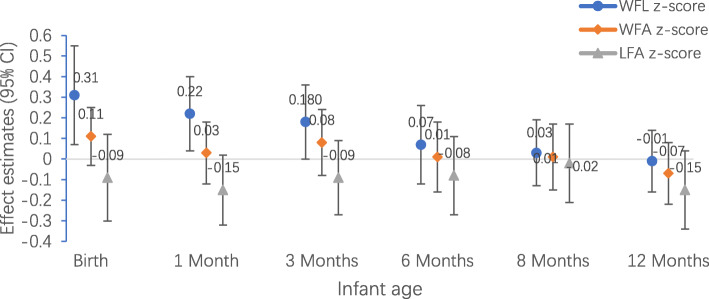
Association of GDM (ref. = non-GDM) with sex-specific WFLZ, WFAZ, FLAZ (β, 95% CI) from birth to 12 months among 945 mother-child pairs. Models are adjusted for, pre-pregnancy BMI, maternal age, parity, gestational age. Abbreviations: GDM, gestational diabetes mellitus; CI, confidence interval; WFLZ, weight-for-length z-score; WFAZ, weight-for-age z-score; LFAZ, length-for-age z-score; BMI, body mass index

Figure 2 shows the results of multivariable regression analysis of GDM with sex-specific WFLZ from birth to 12 months of age in women who were underweight, normal weight, and overweight/obese before pregnancy. Infants born to mothers with GDM had a greater WFLZ at birth [β:0.36 SD units (95% CI: 0.11–0.61)], 1 month [β:0.22 SD units (95% CI: 0.03–0.41)], and 3 months [β:0.19 SD units (95% CI: 0.01–0.37)]. The association was not significant at 6, 8 or 12 months of age, and the effect estimate decreased across infancy. The infants of mothers with GDM had lower LFAZs across infancy, but the difference did not reach significance. GDM status was not significantly associated with LFAZ at any time. In the sensitivity analysis, adjusting for pre-pregnancy BMI did not significantly change the results (Fig. S2).

## Discussion

In this prospective birth cohort study, maternal GDM status was positively associated with infant WFLZ scores. The associations were more pronounced in normal and overweight/obese women than in underweight women. The effect estimates of the association between GDM and infant WFLZ scores was highest at birth and decreased across infancy until 12 months of age. The results may help to identify the critical period and the specific risk groups of GDM for childhood weight status.

The results are consistent with previous studies that reported a positive correlation of maternal hyperglycemia during pregnancy and infant body weight [43, 44]. A large cohort study in Finland reported that offspring of GDM mothers were more likely to be overweight at 5, 7, and 12 years of age, and a multinational cross-sectional study found that the odd ratios for obesity (OR = 1.53) and central obesity (OR = 1.73) were higher in offspring of mothers with GDM compared to those without GDM [44]. A previous meta-analysis also found that maternal diabetes was associated with increased infant fat mass and body fat percentage [45]. However, other observational studies failed to find positive associations between GDM and offspring weight status [6, 21]. Additional randomized, controlled trials or intervention studies are needed to provide high-quality evidence. It has been proposed that maternal hyperglycemia leads to excess fetal insulin that alters the expression of hypothalamic neurotransmitters and leads to childhood hyperphagia and overweight [46]. Mothers with GDM may share metabolic, genetic, and behavioral risk factors with their children that promote the development of obesity.

Pre-pregnancy weight influences the risk of both GDM and offspring obesity, however a large sample study (*n* = 14,881) indicated that the association between GDM and childhood obesity was no longer significant after adjusting for the pre-pregnancy BMI [5]. In this study, GDM was associated with an increased infant WFLZ after adjusting for maternal BMI and other confounders, indicating an independent association with infant body weight. Stratification of mothers by BMI resulted in a significant association of GDM and WFLZ in overweight/obese and normal-weight women, but not in underweight women. Potential mechanisms, may involve GDM mothers who are underweight and have low triglyceride and cholesterol levels despite having increased glucose levels, which would provide a balanced intrauterine nutritional environment for the fetus [47, 48].

In this study, the effect estimate decreased across infancy and was not significant after 6 months of age. The influence of GDM on infant weight status may vary with age. A previous study reported that children of mothers with GDM had increased weights at birth and at school age but not at 1–3 years of age [49]. In a study of Pima Indians, the association between GDM and childhood weight status was significant after 9 years of age and not before [50]. Perhaps GDM influences childhood lean and fat body masses differently during early developmental ages. In Project Viva, Oken et al. reported that GDM was associated with an increased sum of skinfold thicknesses (a direct measure of adiposity) but not with BMI, which comprises both lean and fat masses [43]. The results of this study are consistent with a previous Chinese cohort study that reported a significant association between maternal glucose concentration and WFLZ scores at birth but not later [35]. The rate of rapid weight gain in the first 6 months of life is usually slower in infants born to mothers without GDM than in those with GDM, which may explain the loss of significance [3, 35]. Breastfeeding may also weaken the influence of GDM on childhood weight status, and is considered to protect against childhood obesity [51]. Additional study is needed to investigate the effect of GDM on the long-term growth trajectory and risk of obesity.

The study strengths include its population-based prospective design, and objective measurement of maternal blood glucose levels. Repeated standard measures of infant growth at multiple times allows observation of the trend of the effect of GDM on offspring weight status across infancy. Subgroup analysis helps to define target GDM populations for interventions to prevent infant obesity. The study also has several limitations. First, our study participants were from urban areas and had relatively high socioeconomic status, which may explain the high percentage of women with GDM in the study population. Second, the study did not include measures of infant body composition such as fat mass, but that is planned in the long-term follow-up of the children in this cohort. Third, the study focused on childhood weight status of during infancy. There was a trend in the decrease in the estimated effect, but previous studies have reported that the effect may rebound later in childhood [49]. We were not able to observe that in this study, but follow-up is ongoing. Fourth, the loss to follow-up may have resulted in bias even though missing data did not change the significance of the study results. Fifth, the self-reported pre-pregnancy weight status we used is subject to recall bias. However, a previous study found that self-reported pre-pregnancy weight did reflect a woman’s pre-pregnancy weight status when BMI was grouped with categorical variables [52]. Lastly, there may be residual confounding which was not accounted for in this analysis.

## Conclusions

Maternal GDM status was associated with infant WFLZ, but not WFAZ or LFAZ. The association between GDM status and offspring WFLZ was more pronounced in early infancy and among normal-weight and overweight/obese women. Increased public health efforts to prevent GDM in normal-weight and overweight/obese pre-pregnancy mothers are recommended to control offspring overweight or obesity.

## Supplementary Information

**Additional file 1: Table S1.** Characteristics of mothers and infants according to infant overweight/obesity status at 1 year of age.

**Additional file 2: Table S2.** Association between GDM status and blood glucose level and infant BMIZ in a linear mixed effects model.

**Additional file 3: Table S3.** Association between GDM status and blood glucose level and infant growth in linear mixed effects model including 505 mothers and children.

**Additional file 4: Figure S1.** Association between GDM (ref. =non-GDM) with infant sex-specific WFLZ (β, 95% CI) from birth to 12 months of age adjusted for pre-pregnancy BMI, and stratified by pre-pregnancy weight status.

**Additional file 5: Figure S2.** Association of GDM (ref. =non-GDM) with sex-specific WFLZ, WFAZ, FLAZ (β, 95% CI) from birth to 12 months of age and adjusted for maternal pre-pregnancy BMI.

**Additional file 6.** The relative questionnaire items used in Born in Shenyang Cohort Study during middle pregnancy visit.

## Data Availability

The study data could be provided on the request from co-authors.

## Abbreviations

BMI: Body mass index
BMIZ: Body mass index z-score
BISCS: Born in Shenyang Cohort Study
CI: Confidence interval
CNY: Chinese Yuan
GDM: Gestational diabetes mellitus
LME: Linear mixed effects
LFAZ: Length-for-age z-scores
OGTT: Oral glucose tolerance test
OR: Odds ratio
SD: Standard unit
WFAZ: Weight-for-age z-scores
WFLZ: Weight-for-length z-scores

## References

[1] NCD Risk Factor Collaboration (NCD-RisC) (2017). Worldwide trends in body-mass index, underweight, overweight, and obesity from 1975 to 2016: a pooled analysis of 2416 population-based measurement studies in 128·9 million children, adolescents, and adults. Lancet..

[2] Symonds ME, Mendez MA, Meltzer HM, Koletzko B, Godfrey K, Forsyth S (2013). Early life nutritional programming of obesity: mother-child cohort studies. Ann Nutr Metab.

[3] HAPO Study Cooperative Research Group (2009). Hyperglycemia and adverse pregnancy outcome (HAPO) study: associations with neonatal anthropometrics. Diabetes..

[4] Aris IM, Soh SE, Tint MT, Liang S, Chinnadurai A (2014). Effect of maternal Glycemia on neonatal adiposity in a multiethnic Asian birth cohort. J Clin Endocrinol Metab.

[5] Gillman MW, Rifas-Shiman S, Berkey CS, Field AE, Colditz GA (2003). Maternal gestational Diabetes, birth weight, and adolescent obesity. Pediatrics..

[6] Kim SY, England JL, Sharma JA, Njoroge T (2011). Gestational Diabetes mellitus and risk of childhood overweight and obesity in offspring: a systematic review. Exp Diabetes Res.

[7] Woo BJ, Locks LM, Cheng ER, Blake-Lamb TL, Perkins ME, Taveras EM (2016). Risk factors for childhood obesity in the first 1,000 days. Am J Prev Med.

[8] Baptiste-Roberts K, Nicholson WK, Wang NY, Brancati FL (2012). Gestational Diabetes and subsequent growth patterns of offspring: the National Collaborative Perinatal Project. Matern Child Health J.

[9] Boerschmann H, Pfluger M, Henneberger L, Ziegler AG, Hummel S (2010). Prevalence and predictors of overweight and insulin resistance in offspring of mothers with gestational Diabetes mellitus. Diabetes Care.

[10] Chandler-Laney PC, Bush NC, Granger WM, Rouse DJ, Mancuso MS (2012). Overweight status and intrauterine exposure to gestational diabetes are associated with children's metabolic health. Pediatr Obes.

[11] Deierlein AL, Siega-Riz AM, Chantala K (2011). The association between maternal glucose concentration and child BMI at age 3 years. Diabetes Care.

[12] Hummel S, Much D, Rossbauer M, Ziegler AG, Beyerlein A (2013). Postpartum outcomes in women with gestational diabetes and their offspring: POGO study design and first-year results. Rev Diabet Stud.

[13] Kubo A, Ferrara A, Windham GC, Greenspan LC, Deardorff J, Hiatt RA (2014). Maternal hyperglycemia during pregnancy predicts adiposity of the offspring. Diabetes Care.

[14] Lee H, Jang HC, Park HK (2007). Early manifestation of cardiovascular disease risk factors in offspring of mothers with previous history of gestational diabetes mellitus. Diabetes Res Clin Pract.

[15] Lewis KL, Castrucci BC, Gossman G, Mirchandani G, Sayegh MA, Moehlman C (2010). Overweight among low-income Texas preschoolers aged 2 to 4 years. J Nutr Educ Behav.

[16] Lindsay RS, Nelson SM, Walker JD, Greene SA, Milne G, Sattar N (2010). Programming of adiposity in offspring of mothers with type 1 Diabetes at age 7 years. Diabetes Care.

[17] Rijpert M, Evers IM, de Vroede MA, de Valk HW, Heijnen CJ, Visser GH (2009). Risk factors for childhood overweight in offspring of type 1 diabetic women with adequate glycemic control during pregnancy: Nationwide follow-up study in the Netherlands. Diabetes Care.

[18] Kvehaugen AS, Andersen LF, Staff AC (2010). Anthropometry and cardiovascular risk factors in women and offspring after pregnancies complicated by preeclampsia or diabetes mellitus. Acta Obstet Gynecol Scand.

[19] Landon MB, Rice MM, Varner MW, Casey BM, Reddy UM, Wapner RJ (2015). Mild gestational Diabetes mellitus and long-term child health. Diabetes Care.

[20] Pettitt DJ, McKenna S, McLaughlin C, Patterson CC, Hadden DR, McCance DR (2010). Maternal glucose at 28 weeks of gestation is not associated with obesity in 2-year-old offspring: the Belfast hyperglycemia and adverse pregnancy outcome (HAPO) family study. Diabetes Care.

[21] Pham MT, Brubaker K, Pruett K, Caughey AB (2013). Risk of childhood obesity in the toddler offspring of mothers with gestational Diabetes. Obstet Gynecl.

[22] Whitaker RC, Pepe MS, Seidel KD, Wright JA, Knopp RH (1998). Gestational Diabetes and the risk of offspring obesity. Pediatrics..

[23] Hamilton ER, Teitler JO, Reichman NE (2011). Mexican American Birthweight and child overweight: unraveling a possible early life course health transition. J Health Soc Behav.

[24] Lawlor DA, Fraser A, Lindsay RS, Ness A, Dabelea D, Catalano P (2010). Association of existing diabetes, gestational diabetes and glycosuria in pregnancy with macrosomia and offspring body mass index, waist and fat mass in later childhood: findings from a prospective pregnancy cohort. Diabetologia..

[25] Michael M, Justin C, Louise S, Elizabeth S, Shayne T, Heather D (2011). Obesity and type 2 diabetes mellitus in a birth cohort of first nation children born to mothers with pediatric-onset type 2 diabetes. Pediatr Diabetes.

[26] Gillman MW, Oakey H, Baghurst PA, Volkmer RE, Robinson JS, Crowther CA (2010). Effect of treatment of gestational Diabetes mellitus on obesity in the next generation. Diabetes Care.

[27] Crume TL, Ogden L, Daniels S, Hamman RF, Norris JM, Dabelea D (2011). The impact of in utero exposure to Diabetes on childhood body mass index growth trajectories: the EPOCH study. J Pediatr.

[28] Page KA, Romero A, Buchanan TA, Xiang AH (2014). Gestational Diabetes mellitus, maternal obesity, and adiposity in offspring. J Pediatr.

[29] Zhao P, Liu E, Qiao Y, Katzmarzyk PT, Chaput JP, Fogelholm M (2016). Maternal gestational diabetes and childhood obesity at age 9-11: results of a multinational study. Diabetologia..

[30] Hedderson M, Ehrlich S, Sridhar S, Darbinian J, Moore S, Ferrara A (2012). Racial/ethnic disparities in the prevalence of gestational diabetes mellitus by BMI. Diabetes Care.

[31] Makgoba M, Savvidou MD, Steer PJ (2012). An analysis of the interrelationship between maternal age, body mass index and racial origin in the development of gestational diabetes mellitus. BJOG..

[32] Hu J, Oken E, Aris IM, Lin PD, Ma Y, Ding N (2019). Dietary patterns during pregnancy are associated with the risk of gestational Diabetes mellitus: evidence from a Chinese prospective birth cohort study. Nutrients..

[33] Wei Y, Yang H, Zhu W, Yang H, Li H, Yan J, Zhang C (2014). International Association of Diabetes and Pregnancy Study Group criteria is suitable for gestational diabetes mellitus diagnosis: further evidence from China. Chin Med J (Engl).

[34] Wei YM, Yang HX (2011). Comparison of the diagnostic criteria for gestational diabetes mellitus in China. Zhonghua Fu Chan Ke Za Zhi.

[35] Liu G, Li N, Sun S, Wen J, Lyu F, Gao W (2014). Maternal OGTT glucose levels at 26-30 gestational weeks with offspring growth and development in early infancy. Biomed Res Int.

[36] Zhao YL, Ma RM, Zhang Y, Mo YX, Chen Z, Sun YH (2016). Growth patterns of appropriate for gestational age infants of gestational diabetic mothers during the first year. Zhonghua Yi Xue Za Zhi.

[37] Bianco ME, Josefson JL (2019). Hyperglycemia during pregnancy and long-term offspring outcomes. Curr Diab Rep.

[38] Dugas C, Perron J, Kearney M, Mercier R, Tchernof A, Marc I, Weisnagel SJ, Robitaille J (2017). Postnatal prevention of childhood obesity in offspring prenatally exposed to gestational Diabetes mellitus: where are we now?. Obes Facts.

[39] Hu J, Aris IM, Oken E, Yanan M, Ning D, Ming G (2019). Association of Total and Trimester-Specific Gestational Weight Gain Rate with early infancy weight status: a prospective birth cohort study in China. Nutrients..

[40] Diabetes IA, Panel PSGC (2010). International Association of Diabetes and Pregnancy Study Groups Recommendations on the diagnosis and classification of hyperglycemia in pregnancy. Diabetes Care.

[41] WHO Multicentre Growth Reference Study Group (2006). WHO child growth standards based on length/height, weight and age. Acta Paediatr Suppl.

[42] Zhou BF (2002). Cooperative meta-analysis Group of the Working Group on obesity in China, predictive values of body mass index and waist circumference for risk factors of certain related diseases in Chinese adults: study on optimal cut-off points of body mass index and waist circumference in Chinese adults. Biomed Environ Sci.

[43] Wright CS, Rifas-Shiman SL, Rich-Edwards JW, Taveras EM, Gillman MW, Oken E (2008). Intrauterine exposure to gestational Diabetes, child adiposity, and blood pressure. Am J Hypertens.

[44] Hakanen T, Saha MT, Salo MK, Nummi T, Harjunmaa U, Lipi ä inen L, Vuorela N. Mothers with gestational diabetes are more likely to give birth to children who experience early weight problems. Acta Paediatr 2016;105:1166–1172. 10.1111/apa.13468 27170551

[45] Karen ML, Chris G, Matthew J, Shalini S, Neena M (2017). Diabetes in pregnancy and infant adiposity: systematic review and meta-analysis. Arch Dis Child Fetal Neonatal Ed.

[46] Plagemann A, Harder T, Melchior K, Rake A, Rohde W (1999). Elevation of hypothalamic neuropeptide Y-neurons in adult offspring of diabetic mother rats. Neuroreport..

[47] Di Cianni G, Miccoli R, Volpe L, Lencioni C, Ghio A, Giovannitti MG (2005). Maternal triglyceride levels and newborn weight in pregnant women with normal glucose tolerance. Diabet Med.

[48] Sánchez-García A, Rodríguez-Gutiérrez R, Saldívar-Rodríguez D, Guzmán-López A, Mancillas-Adame L (2020). Early triglyceride and glucose index as a risk marker for gestational diabetes mellitus. International journal of gynaecology and obstetrics: the official organ of the International Federation of Gynaecology and Obstetrics. Int J Gynaecol Obstet.

[49] Silverman BL, Rizzo T, Green OC, Cho NH, Winter RJ, Ogata ES (1999). Long-term prospective evaluation of offspring of diabetic mothers. Diabetes..

[50] Pettitt DJ, Baird HR, Aleck KA, Bennett PH, Knowler WC (1983). Excessive obesity in offspring of Pima Indian women with diabetes during pregnancy. N Engl J Med.

[51] Wender-Ożegowska (2019). Role of gestational weight gain, gestational diabetes, breastfeeding, and hypertension in mother-to-child obesity transmission. Pol Arch Intern Med.

[52] Thomas DM, Oken E, Rifas-Shiman SL, Téllez-Rojo M, Just A, Svensson K (2019). Do women know their Prepregnancy weight?. Obesity (Silver Spring).

