# Supplementary material for: Association of gestational diabetes mellitus with offspring weight status across infancy: a prospective birth cohort study in China
Source: BMC Pregnancy Childbirth. 2021 Jan 6;21:21. doi: 10.1186/s12884-020-03494-7 (PMC7789150; doi:10.1186/s12884-020-03494-7)
Supplement: Supplementary file 1 — Additional file 1: Table S1. Characteristics of mothers and infants according to infant overweight/obesity status at 1 year of age. [file 12884_2020_3494_MOESM1_ESM.docx]

| **Table S1.** Characteristics of mothers and infants according to infant overweight/obesity status at 1 year of age | | | |  |
| --- | --- | --- | --- | --- |
| **Characteristics** | **Infant overweight/obesity,**  **Mean ± SD or n (%)** | | |  |
|  | **No** | **Yes** | **P-value** |  |
|  | **n=768 (94%)** | **n=52 (6%)** |  |  |
| Age at enrollment (Years) | 30 ± 4 | 30 ± 3 | 0.65 |  |
| Ethnicity |  |  | 0.98 |  |
| Han | 636 (93.7) | 43 (6.3) |  |  |
| Others | 132 (93.6) | 9 (6.4) |  |  |
| Educational attainment |  |  | 0.34 |  |
| Middle school or below | 62 (92.5) | 5 (7.5) |  |  |
| High school | 113 (94.2) | 7 (5.8) |  |  |
| College | 524 (93.1) | 39 (6.9) |  |  |
| Graduate or above | 69 (98.6) | 1 (1.4) |  |  |
| Household income per year, CNY |  |  | 0.73 |  |
| <30,000 | 207 (94.5) | 12 (5.5) |  |  |
| 30,000-<50,000 | 205 (93.2) | 15 (6.8) |  |  |
| 50,000-<70,000 | 165 (92.2) | 14 (7.8) |  |  |
| ≥70,000 | 191 (94.6) | 11 (5.5) |  |  |
| Parity |  |  | 0.72 |  |
| 1 | 589 (93.5) | 41 (6.5) |  |  |
| >1 | 179 (94.2) | 11 (5.8) |  |  |
| Gestational age (Weeks) ^a^ | 38.8 ± 1.3 | 38.9 ± 1.3 | 0.63 |  |
| Pre-pregnancy BMI category, kg/m^2^ |  |  | 0.02 |  |
| <18.5 | 120 (97.6) | 3 (2.4) |  |  |
| 18.5-<24.0 | 457 (94.2) | 28 (5.8) |  |  |
| ≥24.0 | 191 (90.1) | 21 (9.9) |  |  |
| Paternal BMI category, kg/m^2 b^ |  |  | 0.45 |  |
| <18.5 | 21 (100.0) | 0 (0.0) |  |  |
| 18.5-<24.0 | 285 (94.1) | 18 (5.9) |  |  |
| ≥24.0 | 433 (93.3) | 31 (6.7) |  |  |
| Infant sex |  |  | 0.03 |  |
| Male | 354 (91.7) | 32 (8.3) |  |  |
| Female | 414 (95.4) | 20 (4.6) |  |  |
| CNY, Chinese Yuan; BMI, body mass index | | | | |

a: n=817 because of missing data; b: n=788 because of missing data.
