# Supplementary material for: Association of gestational diabetes mellitus with offspring weight status across infancy: a prospective birth cohort study in China
Source: BMC Pregnancy Childbirth. 2021 Jan 6;21:21. doi: 10.1186/s12884-020-03494-7 (PMC7789150; doi:10.1186/s12884-020-03494-7)
Supplement: Supplementary file 2 — Additional file 2: Table S2. Association between GDM status and blood glucose level and infant BMIZ in a linear mixed effects model. [file 12884_2020_3494_MOESM2_ESM.docx]

| **Table S2.** Association between GDM status and blood glucose level and infant BMIZ in a linear mixed effects model | | | | |
| --- | --- | --- | --- | --- |
| **Infant Growth Measures** | **GDM (ref.= non-GDM),**  **β (95% CI)** |  | **Blood Glucose Level, β (95% CI)** |  |
|  |  | **Fasting** | **1 h after OGTT** | **2 h after OGTT** |
| BMIZ |  |  |  |  |
| Model 1 ^a^ | 0.23 (0.10, 0.37) | 0.14 (0.04, 0.25) | 0.02 (-0.01, 0.05) | 0.02 (-0.02, 0.06) |
| Model 2 ^a^ | 0.19 (0.06, 0.33) | 0.10 (0.00, 0.21) | 0.01 (-0.02, 0.04) | 0.01 (-0.03, 0.05) |
| Model 3 ^b^ | 0.22 (0.08, 0.35) | 0.13 (0.02, 0.23) | 0.02 (-0.01, 0.05) | 0.02 (-0.02, 0.06) |
| Model 1: adjusted for exact age of infants at each measurement  Model 2: Model 1 + pre-pregnancy BMI | | | | |
| Model 3: Model 2 + maternal age, parity, gestational age | | | | |
| Abbreviations: GDM, gestational diabetes mellitus; CI, confidence interval; OGTT, oral glucose tolerance test; BMIZ, body mass index z-score | | | | |
| a: n=945；b: n=942 because of missing data | | | | |
