# Supplementary material for: Association of gestational diabetes mellitus with offspring weight status across infancy: a prospective birth cohort study in China
Source: BMC Pregnancy Childbirth. 2021 Jan 6;21:21. doi: 10.1186/s12884-020-03494-7 (PMC7789150; doi:10.1186/s12884-020-03494-7)
Supplement: Supplementary file 3 — Additional file 3: Table S3. Association between GDM status and blood glucose level and infant growth in linear mixed effects model including 505 mothers and children. [file 12884_2020_3494_MOESM3_ESM.docx]

| **Table S3.** Association between GDM status and blood glucose level and infant growth in linear mixed effects model including 524 mother-infant pairs | | | | |
| --- | --- | --- | --- | --- |
| **Infant Growth Measures** | **GDM (ref.= non-GDM),**  **β (95% CI)** | **Blood Glucose Level, (95% CI)** | | |
|  |  | Fasting | 1 h after OGTT | 2 h after OGTT |
| WFLZ | 0.29 (0.12, 0.46) | 0.09 (-0.03, 0.22) | 0.02 (-0.02, 0.07) | 0.03 (-0.02, 0.09) |
| WFAZ | 0.18 (0.02, 0.34) | 0.13 (0.01, 0.25) | 0.01 (-0.03, 0.05) | 0.03 (-0.02, 0.08) |
| LFAZ | 0.01 (-0.17, 0.18) | 0.10 (-0.03, 0.22) | 0.00 (-0.04, 0.04) | 0.01 (-0.04, 0.07) |
| Adjusted for exact age of infants at each measurement, pre-pregnancy BMI, maternal age, parity, gestational age | | | | |
| Abbreviations: GDM, gestational diabetes mellitus; CI, confidence interval; OGTT, oral glucose tolerance test; WFLZ, weight-for-length z-score; WFAZ, weight-for-age z-score; LFAZ, length-for-age z-score; BMI, body mass index. | | | | |
