# Supplementary material for: Association of gestational diabetes mellitus with offspring weight status across infancy: a prospective birth cohort study in China
Source: BMC Pregnancy Childbirth. 2021 Jan 6;21:21. doi: 10.1186/s12884-020-03494-7 (PMC7789150; doi:10.1186/s12884-020-03494-7)
Supplement: Supplementary file 4 — Additional file 4: Figure S1. Association between GDM (ref. =non-GDM) with infant sex-specific WFLZ (β, 95% CI) from birth to 12 months of age adjusted for pre-pregnancy BMI, and stratified by pre-pregnancy weight status. [file 12884_2020_3494_MOESM4_ESM.docx]

**Figure S1.** Association between GDM (ref. =non-GDM) with infant sex-specific WFLZ (β, 95% CI) from birth to 12 months of age adjusted for pre-pregnancy BMI, and stratified by pre-pregnancy weight status. Models are adjusted for exact age of infants at each measurement, pre-pregnancy BMI. Abbreviations: GDM, gestational diabetes mellitus; CI, confidence interval; WFLZ, weight-for-length z-score; BMI, body mass index.
