# Supplementary material for: Association of gestational diabetes mellitus with offspring weight status across infancy: a prospective birth cohort study in China
Source: BMC Pregnancy Childbirth. 2021 Jan 6;21:21. doi: 10.1186/s12884-020-03494-7 (PMC7789150; doi:10.1186/s12884-020-03494-7)
Supplement: Supplementary file 5 — Additional file 5: Figure S2. Association of GDM (ref. =non-GDM) with sex-specific WFLZ, WFAZ, FLAZ (β, 95% CI) from birth to 12 months of age and adjusted for maternal pre-pregnancy BMI. [file 12884_2020_3494_MOESM5_ESM.docx]

**Figure S2.** Association of GDM (ref. =non-GDM) with sex-specific WFLZ, WFAZ, FLAZ (β, 95% CI) from birth to 12 months of age and adjusted for maternal pre-pregnancy BMI. Models are adjusted for, pre-pregnancy BMI, maternal age, parity, gestational age. Abbreviations: GDM, gestational diabetes mellitus; CI, confidence interval; WFLZ, weight-for-length z-score; WFAZ, weight-for-age z-score; LFAZ, length-for-age z-score; BMI, body mass index.
