# Supplementary material for: Association of gestational diabetes mellitus with offspring weight status across infancy: a prospective birth cohort study in China
Source: BMC Pregnancy Childbirth. 2021 Jan 6;21:21. doi: 10.1186/s12884-020-03494-7 (PMC7789150; doi:10.1186/s12884-020-03494-7)
Supplement: Supplementary file 6 — Additional file 6. The relative questionnaire items used in Born in Shenyang Cohort Study during middle pregnancy visit. [file 12884_2020_3494_MOESM6_ESM.docx]

**The relative questionnaire items used in Born in Shenyang Cohort Study during middle pregnancy visit**

***Age***

How old are you?

_______ years old.

***Ethnicity***

What is your ethnicity?

_______

***Educational Attainment***

Please choose the highest level of school that you has completed from the following list

□ Primary school

□ Middle school

□ High school

□ College

□ Graduate or above

***Household Income per Year***

Which of the following best describes your household income?

□ <10,000 yuan/year

□ 10,000-<30,000 yuan/year

□ 30,000-<50,000 yuan/year

□ 50,000-<70,000 yuan/ year

□ ≥70,000 yuan/year

***Parity***

The time of natural births of you before this pregnancy

_______ times

The time of cesarean sections of you before this pregnancy

_______ times

***Gestational age***

When was your last menstrual period?

__ __ / __ __ / __ __ (Month / Date / Year)

Today's date

__ __ / __ __ / __ __ (Month / Date / Year)

***Pregnancy’s weight***

How much did you weigh just before this pregnancy?

_________jin (1 jin = 1/2 kilogram)

***Paternal weight***

How much did father of this child weigh now?

_________jins (1 jin = 1/2 kilogram)

***Paternal height***

How tall is the father of this child?

_________centimeters
